# Clinical Characteristics, Treatment, and Short-Term Outcome in Patients with Heart Failure and Cancer

**DOI:** 10.3390/clinpract11040107

**Published:** 2021-12-06

**Authors:** Jędrzej Piotrowski, Małgorzata Timler, Remigiusz Kozłowski, Arkadiusz Stasiak, Joanna Stasiak, Andrzej Bissinger, Dariusz Timler, Wojciech Timler, Michał Marczak, Roman Załuska, Grzegorz Piotrowski

**Affiliations:** 1 Medical Faculty, Medical University of Lodz, 90-419 Lodz, Poland; jpiotr123@wp.pl; 2 Department of Management and Logistics in Healthcare, Medical University of Lodz, 90-419 Lodz, Poland; malgorzata.timler@gmail.com (M.T.); michal.marczak@umed.lodz.pl (M.M.); 3 Department of Emergency Medicine and Disaster Medicine, Medical University of Lodz, 92-212 Lodz, Poland; remigiusz.kozlowski@umed.lodz.pl (R.K.); dariusz.timler@umed.lodz.pl (D.T.); flamorer@gmail.com (W.T.); 4 Department of Physiology, Development and Neuroscience, University of Cambridge, Cambridge CB3 9BB, UK; arek.stasiak@ymail.com; 5 Department of Chemical Engineering and Biotechnology, University of Cambridge, Cambridge CB3 9BB, UK; js744@cam.ac.uk; 6 Cardiooncology Department, Medical University of Lodz, 90-647 Lodz, Poland; andrzej.bissinger@umed.lodz.pl (A.B.); grzegorz.piotrowski@umed.lodz.pl (G.P.); 7 Cardiology Department, Nicolaus Copernicus Memorial Hospital, 93-513 Lodz, Poland

**Keywords:** cancer, heart failure, treatment, short-term outcome

## Abstract

(1) Our study aimed to look at the clinical characteristics, treatment and short-term outcomes of patients hospitalized due to heart failure with coexisting cancer. (2) Methods: Seventy one cancer (Ca) patients and a randomly selected 70 patients without Ca, hospitalized due to heart failure exacerbation in the same time period constituted the study group (Ca patient group) and controls (non-Ca group), respectively. Data on clinical characteristics were collected retrospectively for both groups. (3) Results: Cancer patients presented with a less advanced NYHA class, had more frequent HFpEF, a higher peak troponin T level, and smaller left atrium size, as compared with controls. The in-hospital deaths of Ca patients were associated with: a higher New York Heart Association (NYHA) class, lower HgB level, worse renal function, higher K and AST levels, presence of diabetes mellitus, and HFpEF. By multivariate logistic regression analysis, impaired renal function was the only independent predictor of in-hospital death in Ca patients (OR-1.15; CI 1.05; 1.27); *p* = 0.017). The following covariates entered the regression: NYHA class, HgB, GFR, K^+^, AST, diabetes mellitus t.2, and HFpEF. (4) Conclusions: The clinical picture and the course of heart failure in patients with and without cancer are different.

## 1. Introduction

Improvement in cancer therapy has led to an increasing number of survivors. With the rising average age of cancer patients, the number of those with cardiovascular diseases (CVD) at diagnosis is increasing. About 20% of patients older than 70 years of age with newly diagnosed cancer have coexisting CVD [1]. Surprisingly, patients diagnosed with cancer have a higher prevalence of pre-existing CVD than the general population, and the type and prevalence of CVD varies significantly by cancer aetiology [2]. On the other hand, patients with heart failure (HF) are at a greater risk of cancer occurrence, which increases over time [3,4,5,6]. The incidence of cancer in established HF has been estimated to be in the range of 18.9–33.7 per 1000 person-years by retrospective analyses [7]. Extensive literature is available for the diagnosis and treatment of heart failure due to cancer therapy. Unexpectedly, there is little data on the management of patients with comorbid CVD at diagnosis. Large cardiovascular clinical trials have excluded patients with cancer, whereas oncology trials have excluded patients with CVD. Cardio-oncology guidelines in this field are discussed only in the context of cardiotoxic complications [8]. Therefore, current guidelines do not provide clear instructions on how to manage patients with comorbid conditions with HF and neoplasms. Thus, the clinical manifestation and treatment of cardiac disease in cancer populations is less well known. However, recently, some interest and discussion on this issue appeared in scientific literature [9,10]. In this study, we looked at the clinical characteristics, treatment, and short-term outcomes of patients hospitalized due to heart failure exacerbation and who had also cancer disease. The goal of our paper is to trigger more intensive discussion among the cardiology community on challenging, complex clinical aspects in the population of patients with concomitant cancer and heart failure.

## 2. Materials and Methods

A total number of 71 patients with consecutive cancer history (Ca patients) hospitalized due to the exacerbation of congestive heart failure in our institution between January 2016 and December 2018 were identified retrospectively through discharge cards and included (Group A). A randomly selected 70 patients hospitalized due to heart failure exacerbation in the same time period and without cancer (non-Ca patients) constituted controls (Group B). In order to find possible risk factors of death in Ca patients, two subgroups of Group 1 were analysed: Subgroup A1—Ca patients who died during hospitalization; Subgroup A2—Ca patients who survived. Heart failure was diagnosed according to European Society of Cardiology HF Guidelines criteria [11].

In all patients, we collected: clinical characteristics, NYHA class, HF type (with reduced ejection fraction (HFrEF) or with preserved ejection fraction-HFpEF), aetiology of heart failure, cause of exacerbation, comorbidities, echocardiogram (ECHO) and laboratory findings, treatment, duration of hospitalization, and in-hospital bleedings. GFR was calculated by the Cockroft–Gault method. We also assessed short-term outcomes, such as in-hospital mortality. Ca patients were reviewed for cancer type (solid tumour or blood malignancy), previous, and current radio- and chemotherapy. Reduced ejection fraction was defined as an ejection fraction less than 50% (HFrEF); more or equal 50% was classified as a preserved ejection fraction (HFpEF).

Patients were diagnosed with atrial fibrillation (AF) irrespective of its type (paroxysmal, persistent, chronic). Reliable data on AF occurrence in the past or any ECG recording with AF were needed to confirm an AF diagnosis. All diabetic patients had type 2 diabetes mellitus. Aetiology of HF and the most likely cause for HF exacerbation was determined according to data in discharge charts.

Ischemic aetiology of HF was assumed if the patient had experienced myocardial infarction in the past or coronary angiography revealed significant lesions that were reliably responsible for myocardium damage. Valvular heart disease aetiology of HF was assumed if there was a grade II or higher of mitral or aortic insufficiency and no other plausible cause of HF was present.

In-hospital bleeding was defined as any blood loss identified clinically during hospitalization causing a fall in the haemoglobin level of 1.0 g/dL or more. Troponin T levels were measured in the blood by means of a high-sensitivity test. The study protocol was approved by ethics committee of Medical University of Lodz (permission number RNN/179/17/KE).

### Statistical Analysis

Values were expressed as mean values ± standard deviation. Proportions were given as the number/percentage of persons. Comparison of mean values obtained in 2 groups was performed using the Student’s *t*-test for independent samples. The Shapiro–Wilk test was used to confirm normal distribution of data within the group. If the distribution was not normal, a nonparametric test was used (Mann–Whitney U test). The χ^2^ test for 2 × 2 contingency table with Yates’ correction for continuity was used to evaluate the independence of variables. A Mann–Whitney test was used to determine relations among death, independent characteristics, and chemotherapy. Associations between death and independent variables were assessed by logistic regression analysis. *p* < 0.05 was considered statistically significant. Statistical analysis was performed using Statistica 13.1 program and Microsoft Excel software.

## 3. Results

**Figure 1:**
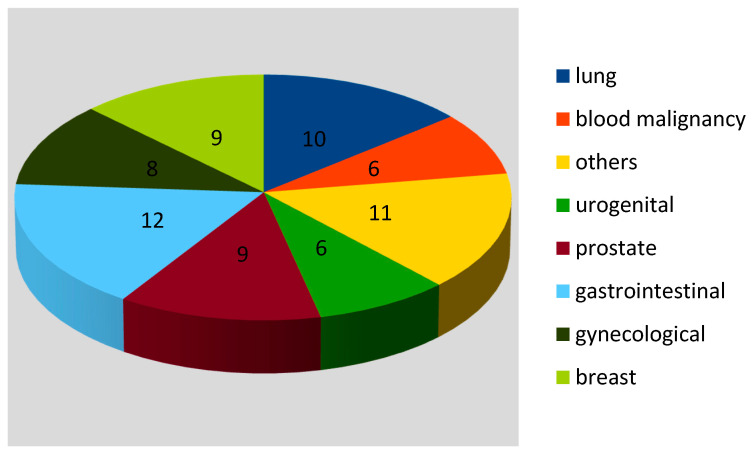
Type of malignancy (number of patients).

Sixty five patients (91.5%) had solid tumours and others had blood malignancies (8.5%). Six patients of the Ca group (subgroup A1) and 2 of the non-Ca group died during hospitalization, which did not make a statistically significant difference (*p* = 0.28). Seventeen (24%) patients underwent chemotherapy and 9 (12.7%) radiotherapy in the past. Current active cancer treatment was noted in only 1 patient. The type of malignancy is presented in Figure 1. Solid cancers were the majority. No Ca patient was in end-stage cancer disease. Data on the cancer status was limited, scarce, and not available in the cardiology ward’s accessible data. For this reason, it was not included in the analysis.

Compared with non-Ca patients, Ca patients presented with a less advanced NYHA class (*p* < 0.001), had more frequent HFpEF (*p* = 0.01), and higher peak troponin T levels (*p* = 0.007). In addition, they had higher EF (*p* = 0.02) and smaller left atrium size (*p* = 0.04). Hypertension was more prevalent in the non-Ca patient group (*p* = 0.03). The hospital course did not differ significantly between the study groups. There were no differences in terms of length of hospital stay (6.17 ± 4.16 vs. 5.92 ± 3.33 days; *p* = 0.35), in-hospital bleedings (2.28% vs. 2.86%; *p* = 0.62), and in-hospital mortality (8.45% vs. 2,86%; *p* = 0.28) between non-Ca and Ca patients, respectively (Table 1).

Cardiac treatment in both groups is summarised in Table 2.

Compared with non-Ca patients, Ca patients were less frequently treated with beta-blockers (*p* = 0.03) and mineralocorticoid receptor antagonists (*p* = 0.01) (Table 2). However, 22 (82%) and 21 (78%) of 27 Ca patients, 39 (87%), and 35 (78%) of 45 non-Ca patients with EF < 40% were on beta-blockers and mineralocorticoid receptor antagonists, respectively, which did not make a statistically significant difference (*p* = 0.74; *p* = 1.0, respectively).

In both study groups, the most common cause of HF was ischemic heart disease (Table 3) and the most frequent factors triggering HF exacerbation were infection and arrhythmia (Table 3).

In Ca patients with HF, by using the Mann–Whitney test, in-hospital death was associated with: a higher NYHA class, lower HgB level, worse renal function, higher K^+^ and AST levels, presence of diabetes mellitus, and HFpEF (Table 4). Chemotherapy was not associated with mortality in cancer patients. In addition, the length of hospital stay (6.5 ± 7.7 vs. 6.1 ± 3.8 days; *p* = 0.31) and in-hospital bleedings (17% vs. 2%; *p* = 0.08) did not differ in Ca patients who died and who survived, respectively.

By multivariate logistic regression analysis, impaired renal function was the only independent predictor of in-hospital death in Ca patients (OR-1.15; CI 1.05; 1.27); *p* = 0.017).

The following covariates entered the regression: NYHA class, HgB, GFR, K^+^, AST, diabetes mellitus t.2, and HFpEF.

We calculated the Mantel–Haenszel (M–H) estimator as an alternative statistical analysis to remove confounding effects.

## 4. Discussion

Heart failure (HF) and cancer are becoming increasingly prevalent as population ages [12]. Both conditions are associated with significant mortality and morbidity. Paradoxically, progress in HF treatment contributes to increasing probability of diagnosing neoplasm and vice versa [3]. To the best of our knowledge, this study is novel in the analysis of clinical characteristics, treatment, and short-term outcome of cancer patients hospitalized in the cardiology ward due to heart failure exacerbation.

Noncardiac comorbidities in patients with HF have been increasingly recognized as factors influencing significant mortality and hospitalizations in HF patients, with the effect being even more pronounced in patients with HFpEF [13]. Cancer as a comorbidity increases mortality in the HF population [3] and particularly concerns the chronic HF setting [10]. Epidemiological data suggest that HF patients who are diagnosed with cancer have higher all-cause mortality than both subjects with HF but no malignancy [3] and cancer patients without HF [5,7]. In addition, cancer patients hospitalized with comorbid HF represent a high-risk population with high inpatient mortality rates [14].

Our study did not reveal higher mortality in cancer patients in an acute HF setting. However, the number of patients was small, and it may be the reason for the lack of statistically significant differences in mortality between cancer and noncancer populations. There are also observations in which trastuzumab-treated women who developed heart failure had better prognoses than matched heart failure controls-patients with no cancer [15].

We found that patients diagnosed with neoplasm and hospitalized due to exacerbation of HF present less advanced symptoms and have a higher prevalence of HFpEF as compared with the general population without cancer. In addition, echocardiography evaluation revealed hearts that were less damaged in patients with cancer and heart failure. Despite this, we found higher levels of troponin T in cancer patients, which may suggest more severe myocardium injury or necrosis. Emergency cancer patients with elevated troponin are at an increased risk of death [16,17]. Trials involving patients with heart failure have shown that detectable troponin, at any level, is associated with a progressive decline in left ventricular systolic function [18]. This is in contrast to our results. Our data does not allow for evaluating exactly why patients with cancer had higher troponin levels despite less myocardium damage. There are a few hypothetic explanations for subtle troponin rise in Ca patients. It may be related to an increased thrombotic risk and, as a consequence, silent myocardial ischemia or undiagnosed acute pulmonary embolism with an underlying acute decompensation of heart failure. A troponin level rise in Ca patients may also reflect residual necrosis as a result of previous chemotherapy that might have triggered the apoptosis of cardiomyocytes [19]. It may be associated with subclinical inflammation, an element of cancer pathology [20,21]. Some patients may have concomitant infections, which triggers a low-grade troponin release.

Furthermore, a positive cardiac troponin test in patients with heart failure exacerbation is associated with higher in-hospital mortality [22]. On the contrary, our findings indicated that in-hospital mortality, as well as hospital course, did not differ regardless of cancer diagnosis and troponin levels. The results of our study showed that the hospital mortality of HF patients with cancer was determined by renal function. Multivariate logistic regression analysis showed that impaired renal function was the only predictor of in-hospital death. We did not find significance in this term for the NYHA class, comorbidities, plasma NT-proBNP, and troponin levels, as well as echocardiographic parameters. Our findings were most similar to other findings, which concluded that the most important prognostic factors among patients with acute HF included low systolic blood pressure and elevated creatinine level [23].

Next, we found that patients with cancer and heart failure were treated with guideline-directed medical therapy, but it was less aggressive as compared with the general population. They were less frequently treated with beta-blockers and mineralocorticoid receptor antagonists. This may be due to the preserved ejection fraction in patients with cancer. The frequency of both beta-blockers and mineralocorticoid receptor antagonists was the same in the treatment of Ca and non-ca patients. In this regard, the results of our study did not differ from other results, which stated that only slightly fewer patients in the general population with HFpEF were treated with diuretics, beta-blockers, MRAs, and ACEIs or ARBs [24]. This may reflect treatment of cardiovascular comorbidities, such as hypertension, which were more frequently observed in our study group without cancer. Some observations suggest that patients with cardiovascular diseases and cancer as a comorbidity are undertreated only because of the neoplasm they suffer from [25]. However, in our study, patients with EF < 40% in both groups were treated with beta-blockers and mineralocorticoid receptor antagonists with the same frequency, and bias against cancer patients was not confirmed by our observations.

Nevertheless, only a minority of cancer patients received optimal medical treatment. Yusuf et al. showed that less than 50% of patients with acute coronary syndrome and cancer received applicable therapy [25]. It may be that comorbidities, cancer status, and cancer therapy affected the physician’s decision [6].Cancer patients were excluded from HF clinical trials with ACE-I, beta-blockers, mineralocorticoid receptors, and statins and we do not have data on their efficacy in the cancer HF population [26]. Although such a therapy saves lives in the general HF population, prospective trials to prove the benefits of this therapy in cancer patients would be unethical. On the other hand, early cardiotoxicity detection (LVEF drop) during chemotherapy and prompt initiation of pharmacological treatment with HF therapy is associated with LVEF recovery and a parallel reduction in cardiac events [27,28].

Finally, our findings indicate that both in general and cancer populations, the main disease leading to heart failure was coronary heart disease. Infection and tachyarrhythmia were the most frequent factors triggering decompensation of heart failure in both groups.

### Study Limitations

This analysis has limitations. First, our studied groups were small despite representing three years of inclusion in our centre. The sample size was insufficient for analysis of specific cancer types. Second, as it is a retrospective observation, some important details are unavailable, including data on cancer staging, which limited analysis and interpretations in some aspects. Third, because of a low number of A1 subgroup patients (*n* = 6), association between renal function and mortality is weak, although in the clinical context, it seems to be obvious.

## 5. Conclusions

In summary, these findings, although novel, should be interpreted with caution. Further studies are needed to answer questions raised by this study.

We have several clinical conclusions. The clinical picture and the course of heart failure in patients with and without cancer are different. Cancer and methods of its treatment may modify the course of HF. Cancer patients with comorbid HF present less advanced symptoms of HF. They seem to have hearts less damaged and be less intensively treated. Nevertheless, the short-term outcome is similar in HF patients with and without cancer. The short-term outcome of HF patients with cancer is predominantly determined by kidney function.

Further assessment and follow-up is needed to perform a more in-depth investigation of the clinical characteristics of HF accompanied by cancer.

## Figures and Tables

**Table 1:** Clinical, laboratory, and echocardiography characteristics of the study groups.

Parameter	Group A (*n* = 71 (%))	Group B (*n* = 70 (%))	*p*-Value
Age	72.03 ± 12.82	71.37 ± 13.71	0.39
Gender (male)	33 (54.93%)	39 (47.14%)	0.36
NYHA	3.06 ± 0.91	3.47 ± 0.5	<0.001
Smoking	16 (22.54%)	12 (17.14%)	0.42
**Comorbidities**			
Hypertension	54 (76.06%)	63 (90.00%)	0.03
Diabetes mellitus	27 (38.03%)	26 (37.14%)	0.91
Dyslipidaemia	33 (46.48%)	33 (47.14%)	0.94
Ischemic heart diseases/myocardial infarction in the past	33 (46.48%)	37 (52.86%)	0.45
Atrial fibrillation	28 (39.44%)	38 (54.29%)	0.08
Chronic kidney disease	23 (32.39%)	30 (42.86%)	0.20
HFpEF	31 (43.66%)	16 (22.86%)	0.01
In-hospital bleedings	2 (2.82%)	2 (2.86%)	0.62
In-hospital mortality	6 (8.45%)	2 (2.86%)	0.28
Duration of hospitalization (days)	6.17 ± 4.16	5.92 ± 3.33	0.35
**Laboratory Parameters**			
Creatinine (mg/dL)	1.25 ± 0.55	1.37 ± 0.71	0.13
GFR (mL/min/m^2^)	50.88 ± 12.47	49.38 ± 13.68	0.25
K^+^ (mEq/L)	4.42 ± 0.64	4.52 ± 0.70	0.19
Na^+^ (mEq/L)	139.24 ± 4.98	136.6 ± 15.34	0.08
HgB (g/dL)	12.51 ± 2.33	13.03 ± 2.05	0.08
WBC (tys/L)	11.08 ± 4.57	9.38 ± 3.69	0.01
PLT (tys/L)	250.79 ± 125.85	217 ± 68.23	0.03
ALT (U/L)	71.27 ± 270.24	81.39 ± 293.31	0.43
AST (U/L)	78.58 ± 235.74	125.74 ± 584.00	0.31
INR	1.81 ± 2.55	1.77 ± 1.40	0.46
TnT (ng/mL)	0.12 ± 0.23	0.05 ± 0.05	0.007
**Echocardiography**			
LVEF (%)	43.08 ± 15.79	37.60 ± 15.33	0.02
LAD (mm)	45.70 ± 7.86	49.12 ± 7.04	0.006
LAV index (mL/m^2^)	58.86 ± 19.69	67.86 ± 24.49	0.04
E/A	1.32 ± 0.81	1.47 ± 0.93	0.25
E/e’	15.95 ± 8.15	22.32 ± 31.77	0.07
NT proBNP (ng/mL)	8725.17 ± 7777.91	7244.95 ± 9570.28	0.31

NYHA—New York Heart Association class, HFpEF—heart failure with preserved ejection fraction, GFR—glomerular filtration rate, K^+^—plasma potassium level, Na^+^—plasma natrium level, HgB—haemoglobin level, WBC—White blood cells count, PLT—Platelet count, ALT—alanine transaminase, AST—aspartate transaminase, INR—international normalised ratio, TnT—troponin T level, NT proBNP—N-terminal pro-B-type natriuretic peptide, LVEF—left ventricle ejection fraction, LAD—left atrium dimension, LAV index—Left Atrium Volume index, E/A—mitral inflow peak early filling velocity to peak atrial filling velocity ratio, E/e’—mitral inflow peak early filling velocity to mitral annular septal peak early diastolic velocity ratio.

**Table 2:** Medical treatment in the study groups.

Medication	Group A (*n* = 71 (%))	Group B (*n* = 70 (%))	*p*-Value
ACE-I/ARA	48 (67.61%)	55(78.57%)	0.35
Beta-blockers	53 (74.65%)	62 (88.57%)	0.03
MRA	35 (49.30%)	50 (71.43%)	0.01
Diuretics (on discharge)	57 (80.28%)	63 (90.0%)	0.11
Ivabradine	3 (4.23%)	3 (4.29%)	0.69
Statin	48 (67.61%)	55 (78.57%)	0.14

ACE-I/ARA—angiotensin-converting enzyme inhibitors/angiotensin II type 1 receptor antagonists; MRA—mineralocorticoid receptor antagonists.

**Table 3:** Aetiologies of heart failure and triggering factors of its exacerbation.

Aetiogy of Heart Failure	Group A (*n* = 71 (%))	Group B (*n* = 70 (%))	*p*-Value
Coronary artery disease	30 (42.25%)	38 (54.29%)	0.12
Hypertension	9 (12.68%)	4 (5.71%)	0.15
Valvular heart disease	10 (14.09%)	8 (11.43%)	0.16
Cardiomyopathy	11 (15.49%)	12 (17.14%)	0.41
Tachyarrhythmias/bradyarrhythmias	7 (9.86%)	12 (17.14%)	0.81
Others	4 (5.63%)	3 (4.29%)	0.21
**Triggering Factor**			
Uncontrolled blood pressure	9 (12.68%)	10 (14.298%)	0.67
Tachyarrhythmia/bradyarrhytmia	27 (38.03%)	20 (28.57%)	0.15
Infection	20 (28.17%)	20 (28.57%)	0.96
Kidney disease exacerbation	2 (2.82%)	3 (4.29%)	0.99
Nonadherence with medications	9 (12.68%)	7 (10%)	0.25
Others	2 (2.82%)	3 (4.29%)	0.28
Unknown	4 (5.63%)	9 (12%)	0.09

**Table 4:** Statistical analysis (Mann–Whitney test and Ch2 test for categorical variables) between independent characteristics and death. Subgroup A1—Ca patients who died during hospitalization. Subgroup A2—Ca patients who survived. The table presents only statistically significant correlations.

Parameter	Subgroup A1 (*n* = 6)	Subgroup A2 (*n* = 65)	*p*-Value
NYHA	3.4 ± 0.2	3.0 ± 0.9	0.008
HgB (g/dL)	10.5 ± 2.5	12.7 ± 2.2	0.045
GFR (mL/min/m^2^)	34 ± 18	53 ± 11	0.008
K^+^ (mEq/L)	5.6 ± 0.9	4.3 ± 0.5	<0.001
AST (U/L)	109 ± 124	74 ± 248	0.021
Diabetes Mellitus type 2	5.0 (83%)	26 (40%)	0.014
HFpEF	5.0 (83%)	26 (40%)	0.031

NYHA—New York Heart Association class, HgB—haemoglobin level, GFR—glomerular filtration rate, K^+^—plasma potassium level; AST—aspartate transaminase, HFpEF—heart failure with preserved ejection fraction.

## Data Availability

De-identified raw data and materials described in the manuscript are freely available from the corresponding author on reasonable request.
